# Perspectives on Low Temperature Tolerance and Vernalization Sensitivity in Barley: Prospects for Facultative Growth Habit

**DOI:** 10.3389/fpls.2020.585927

**Published:** 2020-11-09

**Authors:** María Muñoz-Amatriaín, Javier Hernandez, Dustin Herb, P. Stephen Baenziger, Anne Marie Bochard, Flavio Capettini, Ana Casas, Alfonso Cuesta-Marcos, Claus Einfeldt, Scott Fisk, Amelie Genty, Laura Helgerson, Markus Herz, Gongshe Hu, Ernesto Igartua, Ildiko Karsai, Toshiki Nakamura, Kazuhiro Sato, Kevin Smith, Eric Stockinger, William Thomas, Patrick Hayes

**Affiliations:** ^1^Department of Soil and Crop Sciences, Colorado State University, Fort Collins, CO, United States; ^2^Department of Crop and Soil Science, Oregon State University, Corvallis, OR, United States; ^3^Department of Agronomy and Horticulture, University of Nebraska-Lincoln, Lincoln, NE, United States; ^4^Limagrain Europe, Clermont-Ferrand, France; ^5^Field Crop Development Centre, Alberta Agriculture and Forestry, Lacombe, AB, Canada; ^6^Consejo Superior de Investigaciones Científicas (CSIC), Aula Dei Experimental Station, Zaragoza, Spain; ^7^Bayer – Crop Science, Woodland, CA, United States; ^8^Saatzucht Ackermann GmbH & Co. KG, Irlbach, Germany; ^9^Secobra Recherches, Centre de Bois Henry, Maule, France; ^10^Bavarian State Research Center for Agriculture, Institute for Crop Science, Freising, Germany; ^11^United States Department of Agriculture-Agricultural Research Service (USDA-ARS), Aberdeen, ID, United States; ^12^Department of Molecular Breeding, Center for Agricultural Research, Martonvásár, Hungary; ^13^Division of Field Crops and Horticulture Research Tohoku Agricultural Research Center National Agriculture and Food Research Organization (NARO), Morioka, Japan; ^14^Institute of Plant Science and Resources, Okayama University, Kurashiki, Japan; ^15^Department of Agronomy and Plant Genetics, University of Minnesota, St. Paul, MN, United States; ^16^Department of Horticulture and Crop Science, The Ohio State University/Ohio Agricultural Research and Development Center (OARDC), Wooster, OH, United States; ^17^The James Hutton Institute (JHI), Invergowrie, United Kingdom

**Keywords:** barley, low temperature tolerance, GWAS, meta-analysis, facultative, multi-environments

## Abstract

One option to achieving greater resiliency for barley production in the face of climate change is to explore the potential of winter and facultative growth habits: for both types, low temperature tolerance (LTT) and vernalization sensitivity are key traits. Sensitivity to short-day photoperiod is a desirable attribute for facultative types. In order to broaden our understanding of the genetics of these phenotypes, we mapped quantitative trait loci (QTLs) and identified candidate genes using a genome-wide association studies (GWAS) panel composed of 882 barley accessions that was genotyped with the Illumina 9K single-nucleotide polymorphism (SNP) chip. Fifteen loci including 5 known and 10 novel QTL/genes were identified for LTT—assessed as winter survival in 10 field tests and mapped using a GWAS meta-analysis. *FR-H1*, *FR-H2*, and *FR-H3* were major drivers of LTT, and candidate genes were identified for *FR-H3*. The principal determinants of vernalization sensitivity were *VRN-H1*, *VRN-H2*, and *PPD-H1*. *VRN-H2* deletions conferred insensitive or intermediate sensitivity to vernalization. A subset of accessions with maximum LTT were identified as a resource for allele mining and further characterization. Facultative types comprised a small portion of the GWAS panel but may be useful for developing germplasm with this growth habit.

## Introduction

To meet the challenges of climate change, there has been increasing interest in the agronomic potential of fall-sown barley in northern latitudes (Fisk et al., 2013; Cuesta-Marcos et al., 2015). Although fall-sown barley in regions with winter precipitation patterns can have significant yield advantages compared with spring-sown barley, there is a heightened risk of low temperature-induced crop injury. This risk is problematic at two stages of development: at the vegetative stage, which is the focus of this research, and at flowering. For recent research on low temperature injury at flowering in cereals, please see Trevaskis et al. (2007); Chen et al. (2009), and Powell et al. (2012). Low temperature tolerance (LTT) at the vegetative stage is an inducible trait that involves a complex gene regulon (reviewed by Cuesta-Marcos et al., 2015), and it is a key component of the mega-phenotype known as “winter-hardiness.” Recognizing that a range of factors can determine winter-hardiness, in this report, we will focus on LTT, as measured by winter survival (WS). In this report, we will use LTT and WS synonymously. Additional traits that may contribute to winter-hardiness are sensitivity to vernalization (VRN) (Hayes et al., 1993) and sensitivity to short-day photoperiod (sd-PPD) (Fowler et al., 2001). The sd-PPD phenotype is defined as the case where the recessive “sensitive” (null) allele confers slow or no growth under short-day conditions and the “insensitive” (dominant) allele confers normal, or near-normal, growth rates under short-day conditions. It is difficult to measure VRN sensitivity under field conditions. This trait can be approximated by measuring the days to flowering (DTF) of unvernalized plants under controlled environment conditions and is the approach we follow in this report. Although barley and other cereals are considered long-day plants, there is phenotypic variation in barley for flowering time under short-day conditions; this variation is due to allelic variation at *PPD-H2* (Karsai et al., 2008; Kikuchi et al., 2012). The functional allele allows for flowering under sd-PPD; the non-functional allele delays flowering under sd-PPD. We use “sensitivity” in this context to refer to the non-functional allele. Sensitivity to sd-PPD is a challenge to measure as a phenotype. Under field conditions, the flowering time of fall-planted cereals is determined by multiple genes responding to a range of environmental signals. Sensitivity to sd-PPD can be measured under controlled environment conditions, but the requirement that experimental materials be exposed only to short photoperiod for an extended time makes it challenging to assess the phenotypic responses of larger germplasm arrays. Therefore, potential sensitivity to sd-PPD is most easily assessed based on the allele-specific genotyping of *PPD-H2* (Kikuchi et al., 2012). Understanding the genetics and physiology of LTT, VRN, and sd-PPD will, therefore, lead to more efficient breeding of fall-planted barley and utilization of the growth habit type “facultative.”

Facultative growth habit, as defined by von Zitzewitz et al. (2011), is defined as maximum LTT, sd-PPD sensitivity, and no VRN sensitivity. The theoretical advantage of facultative growth habit in the context of climate change and climate volatility is that it could give growers and processors maximum flexibility because the same variety could be planted in the fall or in the spring. If planted in the fall, the variety would be capable of achieving maximum LTT, and sd-PPD would ensure a timely vegetative to reproductive transition once day length reached a critical threshold. If planted in the spring, the inducible LTT regulon would not be triggered, and thus there would be no cost in reproductive fitness to the variety. Likewise, day length would be sufficiently long that sd-PPD would not be a factor. Genetic keys to the facultative growth habit scenario are the loss-of-function deletion of the *VRN-H2* complex locus, or at least critical elements of it, and the presence of the winter allele in *VRN-H1* with the intact, full intron 1 region (Karsai et al., 2005; von Zitzewitz et al., 2005; Rizza et al., 2016).

There is an extensive literature on the association of VRN sensitivity with LTT (reviewed by Cuesta-Marcos et al., 2015). VRN sensitivity will delay the vegetative to reproductive transition and is therefore a potential mechanism for ensuring maximum LTT. However, a problem with VRN sensitivity in the context of climate change is that it cannot be relied upon to delay the vegetative to reproductive transition: the VRN requirement can be met–and the vegetative to reproductive transition initiated–long before the risk of low temperature injury is past. In contrast, sd-PPD sensitivity should be a much better “insurance” against precocious transition to reproductive growth because climate change is not altering day length. There is no known cost to VRN sensitivity under fall-sown conditions, and there are reported yield advantages whose physiological basis is not clear (Casao et al., 2011a). However, if it can be demonstrated that there is no intrinsic penalty in terms of reproductive fitness, elimination of VRN sensitivity within an overall genome architecture of maximum LTT and sd-PPD sensitivity could be beneficial. Variety development could be streamlined, and commercial production simplified. The breeder could reduce breeding cycle time by bypassing the need for vernalization; the grower could plant the same variety whenever field and/or market conditions were optimum; seed companies could potentially reduce the number of cultivars they need to have available; and the end-user would have the assurance of similar quality/processing attributes, regardless of production season.

There is accumulating evidence that facultative growth habit, as defined, is feasible and viable. Bi-parental quantitative trait locus (QTL) mapping (Fisk et al., 2013) and genome-wide association studies (GWAS) (von Zitzewitz et al., 2011) confirm that maximum LTT is possible with the deletion of *VRN-H2*. Cuesta-Marcos et al. (2015) used near-isogenic lines and a suite of genomics tools to make the same point. A brief review of the key genes discovered in these studies provides essential perspective and framework for the research described in this report. The QTLs and genes (when known) related to LTT are: *FR-H1* (*HvBM5a*) (Fu et al., 2005; von Zitzewitz et al., 2005; Dhillon et al., 2010), *FR-H2* (a cluster of CBF transcription factors) (Skinner et al., 2005, 2006), and *FR-H3* (Fisk et al., 2013). The VRN genes are *VRN-H1* (*HvBM5a*) (Danyluk et al., 2003; Trevaskis et al., 2003; Yan et al., 2003; von Zitzewitz et al., 2005; Cockram et al., 2007; Dhillon et al., 2010), *VRN-H2* (*ZCCT-Ha,b,c*) (Yan et al., 2004), and *VRN-H3* (*HvFT1*) (Yan et al., 2006; Faure et al., 2007; Kikuchi et al., 2009). Sd-PPD is determined by *PPD-H2* (Pan et al., 1994; Fowler et al., 2001; Faure et al., 2007; Cuesta-Marcos et al., 2008; Kikuchi et al., 2009; Casao et al., 2011b; Kikuchi et al., 2012). *PPD-H1* (Karsai et al., 1999; Turner et al., 2005) determines flowering time under long-day conditions and is therefore not directly relevant to facultative growth habit but could be important for facultative varieties under spring-sown conditions. The cited studies were based on a relatively narrow sample of barley germplasm.

To further our understanding of LTT, VRN sensitivity, and facultative growth habit, we assembled a large (*n* = 882) panel of diverse barley germplasm and assessed it for WS under field conditions in a large number of field tests (24 locations over a 2-year period). The panel was also characterized for VRN sensitivity, measured as DTF, under controlled environment conditions. We conducted a GWAS meta-analysis based on Fisher’s test of combined probabilities to integrate WS data from multiple environments. This approach increases the power of detection and reduces false-positive associations (Evangelou and Ioannidis, 2013). A subset of lines were then re-genotyped for targeted alleles at known LTT, VRN, and PPD loci in order to test the predictive utility of the genetic model for facultative growth habit.

## Materials and Methods

### Plant Materials

An array of 882 barley accessions composed of cultivars, landraces, and advanced generation experimental lines contributed by 21 barley breeding and/or genetics programs from around the world was used to evaluate LTT (as WS) and VRN sensitivity (as DTF). Information on the accessions and programs contributing to germplasm is listed in Supplementary Table S1. With the goal of excluding spring accessions that would serve only to confirm the value of alleles at the known major LTT QTLs, a criterion for inclusion in the array was the expectation of some degree of LTT. The largest contributors to the panel were: (1) Oregon State University (OSU) and the University of Minnesota (UM), both United States via the Facwin-6 (Belcher et al., 2015), and (2) the James Hutton Institute (JHI) and the University of Dundee (Scotland, United Kingdom) via the Association Genetics of UK Elite Barley (AGOUEB) (Cockram et al., 2010) and Genomics-Assisted Exploration of Barley Diversity (ExBarDiv) projects (Tondelli et al., 2013).

### Phenotyping

The array (or subsets thereof) was phenotyped for WS under field conditions at 13 locations in 2013–2014 [Canada, France, Germany (two sites), Hungary, Japan, Scotland, Spain, and the United States (five sites)] and 11 locations in 2014–2015 [Canada, France, Germany, Hungary, Scotland, Spain, and the United States (five sites)]. Details on the specific test sites are provided in Supplementary Table S2. Data from the locations that grew the full array were used for this report; data from the locations that grew the subsets of the array were not used. A Type II modified augmented design (Lin and Poushinsky, 1983, 1985) was used at each location. In this design, a single replicate of the accessions was distributed among blocks, each containing three replicated checks. The primary check was “Alba” (winter growth habit), and the secondary checks were “Maja” (facultative growth habit) and “Full Pint” (spring growth habit). Each entry was grown in a single row, 1-m long plot, and WS was assessed visually as the percentage of plants that survived the winter. This design allows entry values to be adjusted based on the primary and secondary checks. Based on the relative efficiency, there was no advantage to either primary or secondary adjustments: therefore, un-adjusted WS values were used for all subsequent analyses.

The array was phenotyped for VRN sensitivity, by measuring DTF, under greenhouse conditions in 2015 at OSU, Corvallis, Oregon (United States). A single unvernalized plant of each accession was grown in a six cell-pack, where each plant had a total soil volume of 85 cm^3^. The Alba, Maja, and Full Pint checks were each replicated 10 times. The greenhouse was maintained at 18 ± 2°C. Natural daylight was supplemented with high intensity lighting to ensure a photoperiod regime of 16 h light/24 h. DTF was recorded for each plant when the first inflorescence was 50% emerged from the boot. The experiment was terminated at 154 days after planting, and any accession that had not flowered was assigned a DTF value of 154. Twenty-one accessions were not included in the VRN phenotyping due to the lack of seed, and three accessions that were included did not germinate. These 24 accessions were not included in subsequent analyses of DTF data. Growth habit classification data (winter, facultative, spring), when available, were obtained for these accessions from USDA-GRIN^1^. Based on DTF, accessions were separated into three groups: those with vernalization sensitivity (DTF ≥ 130), those with vernalization insensitivity (DTF ≤ 73), and those with intermediate sensitivity (DTF ≥ 74 and ≤ 129). A DTF threshold value ≤ 73 for vernalization insensitive germplasm was based on “Dicktoo,” a well-characterized facultative barley (Karsai et al., 2005) and accession 06OR-20, as described in the “Results” section. Inflorescence type (2-row, 6-row) was recorded for each accession. The WS and DTF phenotype data for each accession are provided in Supplementary Table S1.

### Genotyping

The 882 accessions were genotyped with the barley 9K iSelect array (Comadran et al., 2012). Genotyping of the AGOUEB and ExBarDiv germplasm was conducted at TraitGenetics GmbH in Gatersleben, Germany, whereas the remaining germplasm was genotyped at the USDA-ARS Small Grains Genotyping Center in Fargo, ND, United States. After combining all available data and filtering for data quality (>20% missing data) and minor allele frequency (MAF) <0.05, there were 5,725 single-nucleotide polymorphism (SNP) loci assayed on each of 882 accessions. Physical coordinates of iSelect SNPs on the barley reference genome (Mascher et al., 2017) were retrieved from BARLEX^2^ (Colmsee et al., 2015). Of the 5,275 SNPs, 4,875 have an assigned physical position on the reference genome. The complete genotype data are provided in Supplementary Table S3.

A KASP genotyping assay (LGC Genomics, Teddington, United Kingdom) targeting specific loci involved in growth habit and vernalization was performed in a selected subset of 93 accessions based on the first year of field WS data. The targeted loci were *PPD-H1*, *PPD-H2*, *VRN-H1*, and *VRN-H2*. Data are provided in Supplementary Table S4. After a second year of WS data were obtained, 23 of the original 93 accessions chosen in Year 1 were in the overall top 5% based on the average of the 10 environments, across 2 years, where there was differential WS. In this report, we focus on these 23 accessions (Table 3).

### Data Analysis

Principal component analysis (PCA) and linkage disequilibrium (LD) analyses were conducted in TASSEL v.5 (Bradbury et al., 2007) using SNPs with MAF >0.05. LD was estimated as the correlation coefficient *r*^2^ between pairs of SNPs within each chromosome. The decay of LD over physical distance was investigated by plotting pair-wise *r*^2^ values and generating a locally weighted scatterplot smoothing (LOESS) curve. LD decay distance was determined when *r*^2^ fell to the critical threshold estimated from the 99th percentile *r*^2^ distribution for unlinked markers. For GWAS, the mixed-linear model (Zhang et al., 2010) implemented in TASSEL v.5 was used, with a principal component analysis (3 PCs) accounting for population structure in the dataset. For WS, GWAS was first performed on each individual environment. In order to statistically pool GWAS results from the different environments and to detect gene by environment interactions, a meta-analysis was performed (Manning et al., 2011; Kang, 2015). We used Fisher’s method (Fisher, 1925) for combining *p*-values across environments, as follows:

$$X^{2}=-2\,\sum_{i=1}^{k}l\,n\,\left(p\,i\right)$$

where *p*_*i*_ is the *p*-value for the *i*^*th*^ environment. Then, the new Fisher’s *p*-value was calculated using the formula

$$p_{F\,i\,s\,h\,e\,r}=1-Pr\left(X_{2\,k}^{2}\leq X^{2}\right)$$

where $X_{2\,k}^{2}$ is a chi-square variable with *2k* degrees of freedom, and *k* is the number of environments being combined. A false discovery rate (FDR; Benjamini and Hochberg, 1995) threshold of 0.01 was used to identify significant associations. Genes within significant regions were retrieved from the barley reference genome (Mascher et al., 2017) available in BARLEX (see footnote 2).

To assess two locus interactions for VRN sensitivity and WS, a likelihood ratio test (LRT) was performed using a model that includes the two markers tested, their interactions, and population structure based on PCs and a model including only PCs (Cuesta-Marcos et al., 2010). The LRT for VRN sensitivity was based on SNPs with a *q*-value ≤0.1, based on association analysis. The significant SNPs from this analysis were then used to identify the interactions between loci associated with WS. A phylogenetic tree of the 5% accessions with maximum WS (using the average of 10 environments) was generated using the neighbor-joining clustering method implemented in TASSEL v.5.

## Results

### LTT and VRN Sensitivity

**FIGURE 1:**
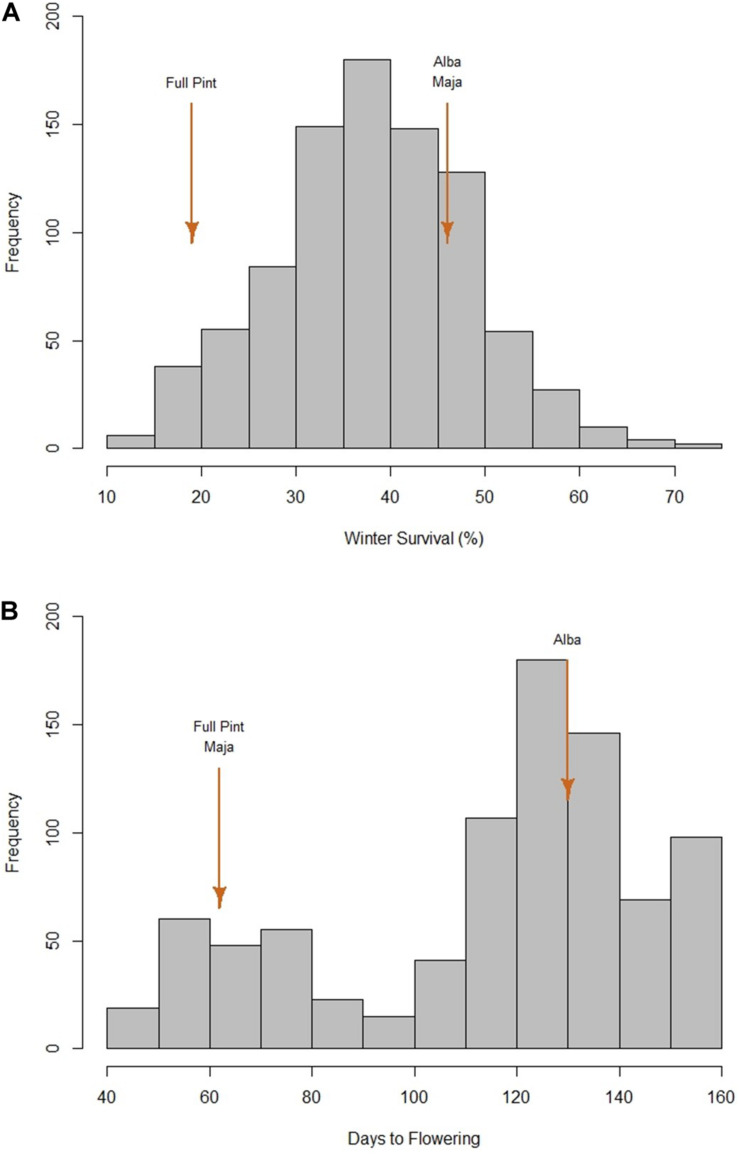
Phenotypic frequency distributions for 882 barley accessions. **(A)** Average winter survival across 10 locations. **(B)** Days to flowering under greenhouse conditions without vernalization. Full Pint, Maja, and Alba are the spring, facultative, and winter checks, respectively.

We used data from the 10 of the 24 environments where there was differential WS: Minnesota (MN) and Ohio (OH) (United States) and Alberta (AB) (Canada) in 2014 and 2015; 2014 Germany (DE); and 2015 Idaho (ID), Scotland (United Kingdom), and Spain (ES). In 13 environments, there was either 100% WS, no detectable difference in WS, or the experiment was compromised in some fashion. In one environment (2014 Nebraska), no entries survived. The frequency distribution of average WS was normal (Figure 1A) and revealed substantial phenotypic variation, with a low of 11% (Mishima 41), a high of 74% (PI87835), and an average of 38%. Considering the checks, the winter (Alba) and facultative (Maja) checks had similar winter survival levels: 46 and 47%, respectively. The spring check (Full Pint) had a WS value of 19%.

The frequency distribution of DTF, without vernalization, clearly separates two groups of accessions, with the dividing point at 95 DTF (Figure 1B). A DTF of 95 under controlled environment conditions, however, is not necessarily predictive of agronomically relevant DTF under field conditions (data not shown). Both Full Pint (the spring check) and Maja (the facultative check) had DTF values of 62, as compared with Alba, the winter check, at 130. In this research, we defined the 139 vernalization-insensitive (spring or potentially facultative) accessions as those with DTF values ≤ 73 based on Dicktoo (DTF = 72) and one accession (O6OR-20) with a DTF of 73. There were 386 accessions with intermediate vernalization sensitivity (≥ 74 and ≤ 129 DTF). The remaining 333 accessions where DTF data were obtained were classified as vernalization sensitive and are defined, for the purposes of this research, as having winter growth habit (Supplementary Table S1).

### LTT and Its Relationship With Test Environments, VRN Sensitivity, and Inflorescence Type

**FIGURE 2:**
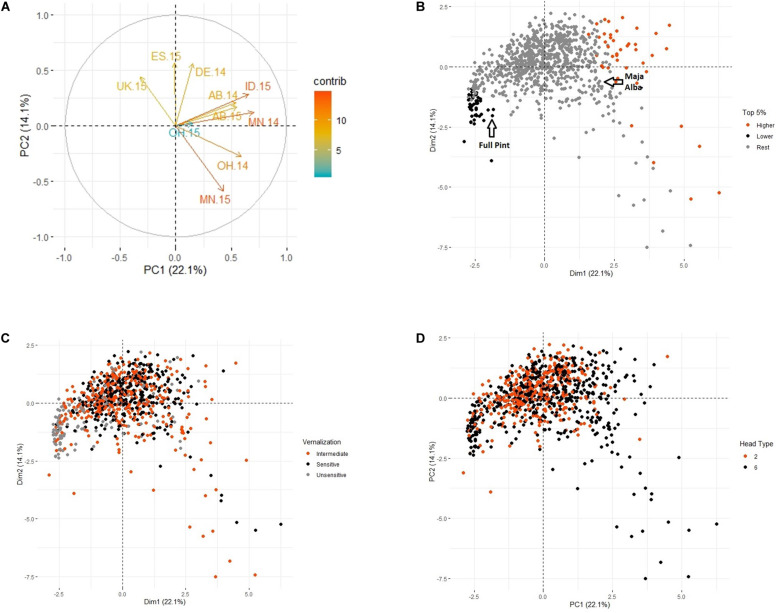
Principal component analysis of average winter survival across 10 locations. **(A)** Contribution of each location to variation across all sites (locations are defined in the text). **(B)** Overlay of the top 5%, bottom 5%, and rest of the accessions for winter survival. **(C)** Overlay of vernalization sensitivity (see text for definitions). **(D)** Overlay of head type (2-row, 6-row).

The relationships between LTT, VRN sensitivity, and spike morphology were assessed using principal component analysis. The first four principal components accounted for 56.6% of the variation for WS. PC1 accounted for 22.1% of the variation, and PC2 accounted for 14.1% of the variation (Figure 2A). The 10 differential environments were clustered into three groups: 1) UK-15, ES-15, and DE-14, 2) OH-15, ID-15, AB-14, AB-15, and MN-14, and 3) OH-14 and MN-15, of which AB-14 and AB-15 were the most similar to each other, and UK-15 was the most unique. The overlay of WS values for the top and bottom 5% of accessions (Figure 2B) showed that accessions with the highest survival values (*n* = 44) were grouped with the Alba and Maja checks, whereas those with the lowest survival (*n* = 44) were grouped with the Full Pint check. The overlay of VRN sensitivity–classified as sensitive, intermediate, and insensitive–showed that most of the VRN-insensitive accessions grouped with Full Pint and the accessions with the lowest LTT, although some VRN-insensitive accessions grouped with the accessions with the highest winter survival. Accessions with intermediate VRN sensitivity, and VRN-sensitive accessions, are found throughput the PCA (Figure 2C). There was a relationship of spike morphology (2-row vs. 6-row) with LTT (Figure 2D): 84% of accessions within the top 5% for LTT were 6-rows. In the full array, there are 529 6-rows and 350 2-rows.

**FIGURE 3:**
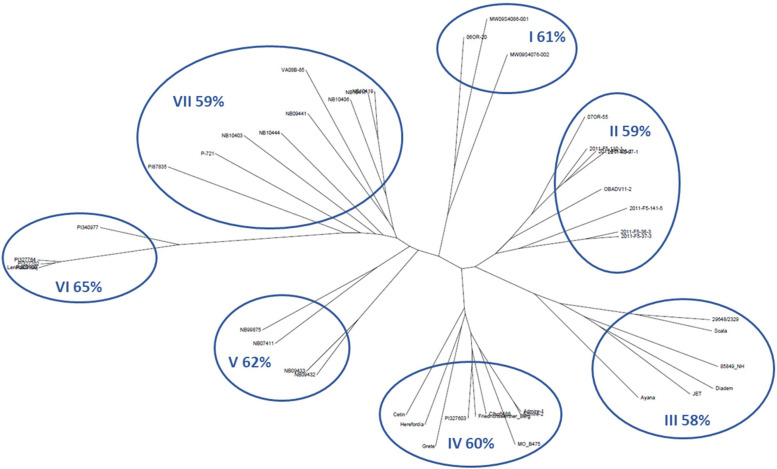
Diversity dendrogram based on SNP genotype data for the top 5% lines of barley accessions for average winter survival across 10 locations. Clades are numbered clockwise, starting with I. The% values are average winter survival for the clade. See Table 1 for more data on the accessions within each clade.

The relationships of LTT, VRN sensitivity, and spike morphology were explored in greater depth using the 5% of accessions with the highest WS across environments. The average WS for this group was 60% (Table 1 and Figure 3). Figure 3 shows an unrooted neighbor-joining dendrogram for these 44 accessions, based on 5,725 SNPs. Each of the seven clades is numbered from the top of the figure, proceeding clockwise, and the average survival of the accessions within each clade is given. The accessions in clade I are all VRN-insensitive 6-rows and trace to the UM or OSU programs. All entries in clade II are 6-rows from the OSU program and are VRN sensitive or are intermediate in their VRN sensitivity. Clade III is composed of 2-rows and 6-rows from European programs, all with winter habit. Clade IV is composed of 6-rows from the US GRIN germplasm collection, of European origin, and accessions contributed by the JHI. All are winters or have intermediate VRN sensitivity, except for Grete, which is VRN insensitive. No DTF data are available for two of the USDA accessions. Clade V is composed of 6-row winter types from the University of Nebraska, Lincoln (UNL) breeding program. Clade VI consists of 6-row germplasm accessions from the USDA collection, all tracing to Russia except for one accession from Armenia. The one accession for which DTF data are available is a winter. Clade VII consists of VRN-sensitive 6-rows from the UNL breeding program, a USDA accession tracing to North Korea, and one accession from the Virginia Polytechnical Institute and State University (VPI-SU) program.

**TABLE 1:** Barley accessions in the top 5% for average winter survival.

**Accession**	**Clade**	**Contributor**	**Source**	**Country of origin**	**Synonym**	**Type**	**Row type**	**DTF**	**Avg. WS**	**Avg. WS clade**
06OR-20	1	OSU-OR	OSU-OR	United States		Breeding line	6	73	58	
MW09S4086-001	1	UMN	UMN	United States		Breeding line	6	69	59	
MW09S4076-002	1	UMN	UMN	United States		Breeding line	6	58	65	61
2011-F5-110-1	2	OSU-OR	OSU-OR	United States		Breeding line	6	139	59	
2011-F5-123-1	2	OSU-OR	OSU-OR	United States		Breeding line	6	146	59	
2011-F5-97-1	2	OSU-OR	OSU-OR	United States		Breeding line	6	130	57	
2011-F5-141-5	2	OSU-OR	OSU-OR	United States		Breeding line	6	127	62	
2011-F5-36-3	2	OSU-OR	OSU-OR	United States		Breeding line	6	154	69	
2011-F5-37-3	2	OSU-OR	OSU-OR	United States		Breeding line	6	126	55	
OBADV11-2	2	OSU-OR	OSU-OR	United States		Breeding line	6	89	58	
07OR-55	2	OSU-OR	OSU-OR	United States		Breeding line	6	82	56	59
29648/2329	3	Lfl	LfL	Germany		Breeding line	2	146	56	
Scala	3	OSU-OR	KWS	Germany		Cultivar	2	152	58	
85849_NH	3	OSU-OR	Secobra	France		Breeding line	2	118	58	
Diadem	3	JHI	AGOUEB	France		Cultivar	6	141	64	
JET	3	JHI	LS Plant Breeding	United Kingdom		Cultivar	2	131	55	
Ayana	3	JHI	AGOUEB	United Kingdom		Cultivar	6	116	58	58
Admire-1	4	OSU-OH	USDA-NSGC	United States	CIho 6377; CI 6377	Germplasm	6	102	65	
Admire-2	4	OSU-OH	USDA-NSGC	United States	CIho 6377; CI 6377	Germplasm	6	152	56	
MO_B475	4	OSU-OH	USDA-NSGC	United States	CIho 9168	Cultivar	6	116	73	
CIho6486	4	OSU-OR	USDA-NSGC	Germany	Berg	Cultivar	6	NA	56	
Friedrichswerther_Berg	4	OSU-OH	USDA-NSGC	Germany	PI174439	Landrace	6	138	56	
PI327603	4	OSU-OR	USDA-NSGC	Czech Republic	Stupicky Sestirady; WIR 18780	Landrace	6	NA	56	
Grete	4	JHI	AGOUEB	Germany		Cultivar	6	60	55	
Herefordia	4	JHI	AGOUEB	?		Cultivar	6	144	57	
Cetin	4	JHI	AGOUEB	Italy		Cultivar	6	118	62	60
NB09432	5	UNL	UNL	United States		Breeding line	6	133	65	
NB09433	5	UNL	UNL	United States		Breeding line	6	135	63	
NB07411	5	UNL	UNL	United States		Breeding line	6	139	56	
NB99875	5	UNL	UNL	United States		Breeding line	6	112	65	62
Leninakanskij	6	OSU-OH	USDA-NSGC	Armenia	PI327669; WIR 19018	Cultivar	6	143	60	
PI327764	6	OSU-OR	USDA-NSGC	Russia	WIR 13829	Landrace	6	NA	68	
PI327702	6	OSU-OR	USDA-NSGC	Russia	WIR 13906	Landrace	?	NA	67	
PI340977	6	OSU-OR	USDA-NSGC	Russia	Donskoj; WIR 19636	Cultivar	6	NA	67	
PI327709	6	OSU-OR	USDA-NSGC	Russia	WIR 14007	Landrace	6	NA	61	65
PI87835	7	OSU-OR	USDA-NSGC	North Korea	Omugi Kauru Pori	Landrace	?	NA	74	
P-721	7	UNL	UNL	United States		Breeding line	6	103	55	
NB10403	7	UNL	UNL	United States		Breeding line	6	137	56	
NB10444	7	UNL	UNL	United States		Breeding line	6	123	59	
NB09441	7	UNL	UNL	United States		Breeding line	6	126	56	
VA08B-85	7	VPI-SU	VPI-SU	United States	Secretariat; PI673931	Breeding line	6	124	55	
NB10406	7	UNL	UNL	United States		Breeding line	6	132	56	
NB10417	7	UNL	UNL	United States		Breeding line	6	128	57	
NB10419	7	UNL	UNL	United States		Breeding line	6	114	62	59

**Clade refers to Figure 3. Contributor codes are defined in the text. DTF, days to flowering without vernalization; Avg. WS, average winter survival for the accession across 10 locations; Avg. WS Clade, average winter survival of the accessions in the clade.**

### GWAS of LTT and VRN Sensitivity

**FIGURE 4:**
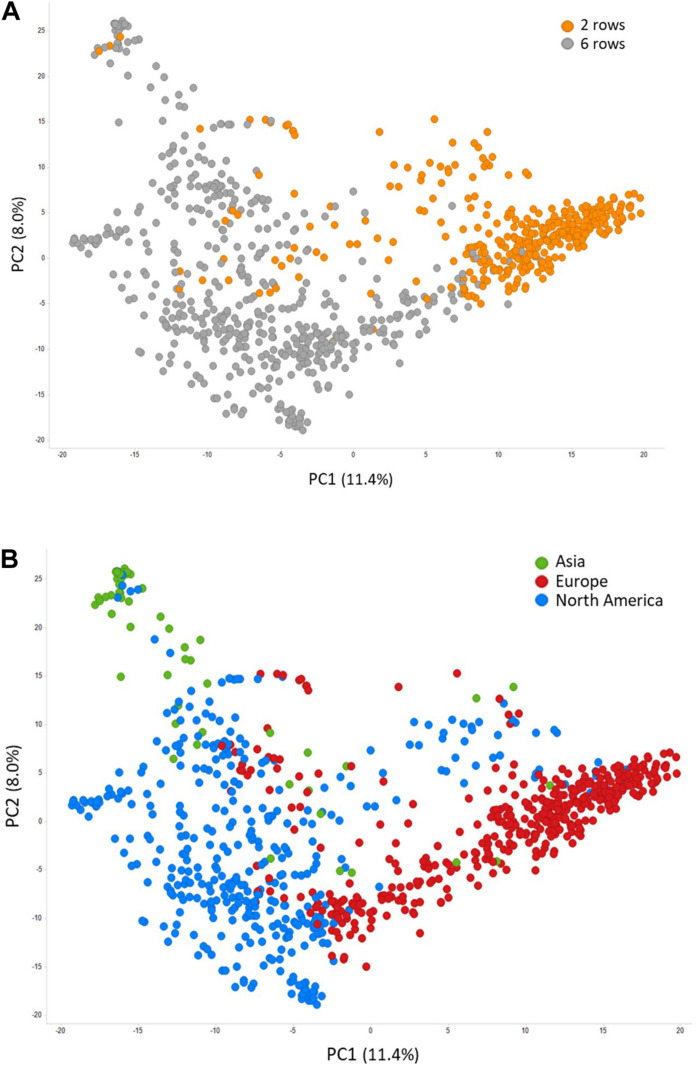
Principal component analysis of 882 barley accessions colored by head type **(A)** and accession origin **(B)**.

Prior to conducting GWAS, we assessed population structure and linkage disequilibrium in the LTT panel of 882 accessions. Structure was evaluated by performing PCA on 5,725 SNPs. As expected, there was strong population structure associated with spike morphology, with the first principal component of the PCA (PC1) mainly separating 2-row from 6-row barley accessions (Figure 4A). Accessions in the first two PCs were largely clustered based on their geographical origin, with PC1 mainly differentiating European from North American barleys, which largely correspond to 2-row and 6-row accessions, respectively, and PC2 separating Asian accessions from the rest (Figure 4B). On a genome-wide level, LD decayed within 24,300 kb (Supplementary Figure S1), although LD varied significantly across the genome, with centromeric and pericentromeric regions generally having higher LD (Supplementary Figure S2). Considering only polymorphic SNPs with a MAF >0.05, there is, on average, one SNP every 793 kb. However, the SNP density is also much higher in more distal, low-LD regions (Supplementary Figure S2).

**FIGURE 5:**
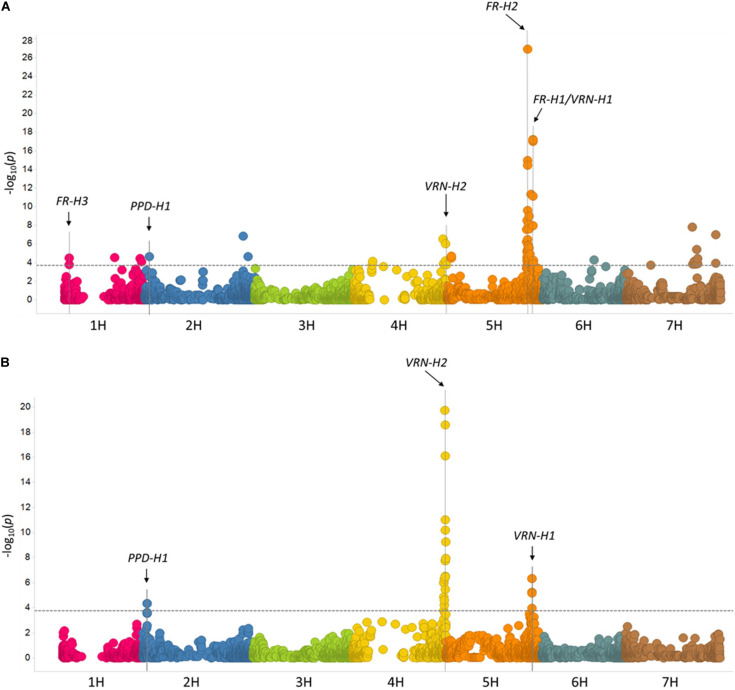
Manhattan plots from the GWAS of 882 accessions. **(A)** Meta-analysis of winter survival. **(B)** Days to flowering without vernalization. -log10 (*p*-values) are shown for 4,875 SNPs with physical coordinates in the barley reference genome (Mascher et al., 2017). The dashed lines indicate the 0.01 FDR-corrected threshold [3.70 for **(A)** and 3.75 for **(B)**].

GWAS of WS was first performed using data from each of the 10 individual environments, and results were then combined via meta-analysis (see section “Materials and Methods” and Supplementary Table S5). A summary of QTL effects across individual environments is shown in Supplementary Table S5. The meta-analysis revealed 15 significant loci on all barley chromosomes except 3H (Figure 5A and Table 2). Five of these loci correspond to previously reported QTLs/genes (*FR-H3*, *PPD-H1*, *VRN-H2*, *FR-H2*, and *FR-H1/VRN-H1*), whereas the other 11 are novel (Figure 5A and Table 2). The major determinants of WS were *FR-H2* and *FR-H1/VRN-H1* (Figure 5A and Table 2), which were also the loci detected in the highest number of individual environments (6/10 environments). Both loci were detected at a very high resolution, with the peak SNP for *FR-H1/VRN-H1* (BOPA1_1501-353) located inside the causative MADS-box transcription factor gene (*HORVU5Hr1G095630*), and the highly significant SNPs on *FR-H2* BOPA2_12_30845 and BOPA2_12_30852 (Supplementary Table S5) contained inside the gene cluster of C-repeat-binding factors (CBFs). *VRN-H2* and the 2H locus with the highest significance level (BOPA2_12_21527; Table 2) were also identified in two individual environments (Supplementary Table S5), whereas another four loci (BOPA2_12_10938 on 1H, *PPD-H1* on 2H, BOPA2_12_10278 on 6H, and BOPA2_12_30645 on 7H; Table 2) were identified in one individual environment (Supplementary Table S5). Each of these eight loci showed gene by environment interactions (Manning et al., 2011; Kang, 2015). The remaining loci, including *FR-H3*, were only identified in the meta-analysis. Three candidate genes for *FR-H3* were identified: *HORVU1Hr1G012690*, which contains the peak SNP (SCRI_RS_114047) and encodes a tetraspanin family protein; *HORVU1Hr1G012680*, which encodes a protein belonging to UDP-glycosyltransferase superfamily; and *HORVU1Hr1G012710*, at 259 kb from the peak SNP, encoding the low temperature-induced protein lt101.2.

**TABLE 2:** Significant peak SNPs associated with winter survival and days to flowering.

**Trait**	**Peak SNP**	**Chr.**	**Pos (bp)**	**(-)Log10 (*p*)**	**Locus**
Winter survival	SCRI_RS_114047	1H	31,685,164	4.48	*FR-H3*
	BOPA2_12_10938	1H	348,204,358	4.54	
	BOPA1_10360-563	1H	526,705,451	4.44	
	BOPA2_12_10880	2H	29,991,973	4.66	*PPD-H1*
	BOPA2_12_21527	2H	687,150,452	6.84	
	SCRI_RS_119513	2H	721,944,874	4.65	
	BOPA2_12_30503	4H	126,859,667	4.12	
	SCRI_RS_226787	4H	619,233,021	6.54	
	BOPA2_12_30873	4H	640,596,465	3.76	*VRN-H2*
	BOPA2_12_10864	5H	30,204,455	4.72	
	SCRI_RS_237352	5H	561,601,170	26.9	*FR-H2*
	BOPA1_1501-353	5H	599,123,281	17.21	*FR-H1/VRN-H1*
	BOPA2_12_10278	6H	356,677,926	4.31	
	BOPA1_8582-772	7H	167,862,263	3.74	
	BOPA2_12_30645	7H	460,352,694	7.79	
	SCRI_RS_233901	7H	624,138,380	7.01	
Days to flowering	BOPA2_12_30871	2H	29,127,021	4.33	*PPD-H1*
	SCRI_RS_142792	4H	639,214,876	19.75	*VRN-H2*
	BOPA1_1501-353	5H	599,123,281	6.33	*VRN-H1*
	BOPA2_12_30930	5H	599,128,110	6.33	*VRN-H1*

**For winter survival, reported SNPs are the result of the GWAS meta-analysis.**

Three loci were significantly associated with DTF: *VRN-H2* on 4H, *VRN-H1* on 5H, and *PPD-H1* on 2H (Figure 5B, Table 2, and Supplementary Table S5). Peak SNPs on *PPD-H1* (BOPA2_12_30871) and *VRN-H1* (BOPA1_1501-353 and BOPA2_12_30930) are within the pseudo-response regulator encoded by *HORVU2Hr1G013400* and the MADS box transcription factor encoded by *HORVU5Hr1G095630*, respectively. As expected, the zinc-finger/CCT domain transcription factor encoded by *VRN-H2* could not be identified by significant SNPs on 4H since the reference genome “Morex” carries a *VRN-H2* deletion.

### Two Locus Interactions

For WS, interactions between VRN sensitivity and QTL/genes associated with LTT were assessed using data from the 6/10 environments where *FR-H2* had a significant effect in the GWAS. Different two-locus combinations were significant at each of the six locations including interactions between *VRN-H2/FR-H2*, *VRN-H1/FR-H2*, and *PPD-H1/FR-H2*. There was no significant *VRN-H1/VRN-H2* interaction in any environment. For VRN sensitivity, highly significant interactions were found between *VRN-H2*, *VRN-H1*, and *PPD-H1* (Supplementary Table S6). The most significant interaction was between *VRN-H1* and *VRN-H2*, and a large -log10 (*p*-value) was also found for the *VRN-H2/PPD-H1* interaction.

### Allele-Specific Genotyping for Growth Habit-Related Genes

The results of the allele-specific genotyping of *PPD-H2*, *PPD-H1*, *VRN-H1*, and *VRN-H2* on accessions with the highest winter survival are shown in Table 3. At the *PPD-H2* locus, three of the VRN-insensitive accessions have the allele conferring sensitivity to sd-PPD, where the plant exhibits delayed flowering under short days. One accession (MW09S4086-001) has the sd-PPD insensitive (functional) allele, where flowering occurs under short-day conditions. Of the remaining VRN intermediate and VRN-sensitive accessions, 11 have the short-day sensitive allele, and 7 have the short-day insensitive allele. A novel deletion at this locus was found in one accession (Leninakanskij). At the *PPD-H1* locus, 22 accessions carry the dominant allele, which promotes flowering under long-day conditions (Turner et al., 2005); only Leninakanskij has the recessive allele, which delays flowering under long-day conditions. All 23 accessions have the “intact” *VRN-H1* winter allele, which is reported to be associated with LTT (Rizza et al., 2016). At *VRN-H2*, two of the four VRN-insensitive accessions have a deletion of all three *ZCCT-H* genes. Two accessions, tracing to the UMN program, have partial deletions at this locus. Complete deletions were observed in two of the accessions with intermediate VRN sensitivity, both from the OSU program. Partial deletions of *VRN-H2* were observed in four accessions, two in the intermediate group (MOB-475 and Cetin) and two in the winter growth habit group (Jet and Diadem).

**TABLE 3:** Allele types at *PPD-H1*, *PPD-H2*, *VRN-H1*, and *VRN-H2* for 23 barley accessions in the top 5% for winter survival across 10 locations.

**Genotype**	**WS**	**DTF**	***PPD-H2* (1H)**	***PPD-H1* (2H)**	***VRN-H1* (5H)**	***VRN-H2* (4H)**
GRETE	55	60	Truncated *HvFT3*	*PPD-H1*	intact intron 1	Deletion *ZCCT_Ha ZCCT_Hb ZCCT_Hc*
MW09S4076-002	65	58	Truncated *HvFT3*	*PPD-H1*	intact intron 1	Deletion *ZCCT_Ha ZCCT_Hb*
06OR-20	58	73	Truncated *HvFT3*	*PPD-H1*	intact intron 1	Deletion *ZCCT_Ha ZCCT_Hb ZCCT_Hc*
07OR-55	56	82	Truncated *HvFT3*	*PPD-H1*	intact intron 1	Deletion *ZCCT_Ha ZCCT_Hb ZCCT_Hc*
OBADV11-2	58	89	Truncated *HvFT3*	*PPD-H1*	intact intron 1	Deletion *ZCCT_Ha ZCCT_Hb ZCCT_Hc*
MW09S4086-001	59	69	Intact *HvFT3*	*PPD-H1*	intact intron 1	Deletion *ZCCT_Ha ZCCT_Hb*
NB09433	63	135	Intact *HvFT3*	*PPD-H1*	intact intron 1	Intact *ZCCT_Ha ZCCT_Hb ZCCT_Hc*
NB10403	56	137	Intact *HvFT3*	*PPD-H1*	intact intron 1	Intact *ZCCT_Ha ZCCT_Hb ZCCT_Hc*
NB10417	57	128	Intact *HvFT3*	*PPD-H1*	intact intron 1	Intact *ZCCT_Ha ZCCT_Hb ZCCT_Hc*
CETIN	62	118	Truncated *HvFT3*	*PPD-H1*	intact intron 1	Partial deletion
DIADEM	64	141	Truncated *HvFT3*	*PPD-H1*	intact intron 1	Deletion *ZCCT_Hc*
JET	55	131	Truncated *HvFT3*	*PPD-H1*	intact intron 1	Partial deletion
NB09432	65	133	Intact *HvFT3*	*PPD-H1*	intact intron 1	Intact *ZCCT_Ha ZCCT_Hb ZCCT_Hc*
LENINAKANSKIJ	60	143	NA	*ppd-H1*	intact intron 1	Intact *ZCCT_Ha ZCCT_Hb ZCCT_Hc*
NB09441	57	126	Intact *HvFT3*	*PPD-H1*	intact intron 1	Intact *ZCCT_Ha ZCCT_Hb ZCCT_Hc*
NB10419	62	114	Intact *HvFT3*	*PPD-H1*	intact intron 1	Intact *ZCCT_Ha ZCCT_Hb ZCCT_Hc*
P-721	55	103	Intact *HvFT3*	*PPD-H1*	intact intron 1	Intact *ZCCT_Ha ZCCT_Hb ZCCT_Hc*
2011-F5-141-5	62	127	Truncated *HvFT3*	*PPD-H1*	intact intron 1	Intact *ZCCT_Ha ZCCT_Hb ZCCT_Hc*
2011-F5-36-3	69	154	Truncated *HvFT3*	*PPD-H1*	intact intron 1	Intact *ZCCT_Ha ZCCT_Hb ZCCT_Hc*
NB07411	56	139	Truncated *HvFT3*	*PPD-H1*	intact intron 1	Intact *ZCCT_Ha ZCCT_Hb ZCCT_Hc*
NB10444	59	123	Truncated *HvFT3*	*PPD-H1*	intact intron 1	Intact *ZCCT_Ha ZCCT_Hb ZCCT_Hc*
NB99875	65	112	Truncated *HvFT3*	*PPD-H1*	intact intron 1	Intact *ZCCT_Ha ZCCT_Hb ZCCT_Hc*
MO_B475	72	116	Truncated *HvFT3*	*PPD-H1*	intact intron 1	Partial deletion

**WS, winter survival; DTF, days to flowering.**

## Discussion

### The Challenges of Phenotyping LTT and VRN Sensitivity

There are advantages and disadvantages to assessing LTT and VRN sensitivity under field and/or controlled environment conditions. Assessment of a GWAS panel for LTT is feasible only under field conditions, but naturally occurring low temperature events are rare, and variables besides low temperature can determine survival. In this study, fewer than half of the test sites provided useful data for GWAS of LTT, and yet every test site has, at some point in the past 30 years, generated useful data for mapping the genetic determinants of this phenotype. The implication is that a large number of test sites need to be sampled in order to ensure a reasonable chance of obtaining useful field data. Therefore, some future research could be directed at smaller subsets of informative germplasm, such as the top 5% identified in this panel. At each field site, perhaps selected based on the probability of generating representative differential survival (e.g., Figure 2A), assessment of WS using replicated large plots is recommended. For example, there was 100% mortality in the GWAS panel single rows at the Nebraska test site in 2014. The nine accessions that were also included in an adjacent replicated yield trial with four, 3 m row plots had WS values ranging from 40 to 81% (Baenziger, personal communication). Furthermore, we recommend that comprehensive weather data be obtained at each site. These multi-location data, coupled with genotype and phenotype data, will be useful in validating and enhancing climate models, such as the one recently reported by Byrns et al. (2020). Smaller subsets would also be amenable to controlled environment tests, where functional genomics approaches can complement GWAS (Fowler et al., 2001; Stockinger et al., 2007).

VRN sensitivity is straightforward to measure as DTF under controlled environment conditions, but the data are only an approximation of DTF under field conditions, where there are complex interactions involving changing photoperiod duration, diurnal fluctuations in temperature, and other environmental signals affecting plant growth and development. A deeper understanding of VRN sensitivity can be obtained under field conditions with repeated plantings from fall to spring (Igartua et al., 1999) and in controlled environment tests involving factorial combinations of time, temperature, and photoperiod duration (Casao et al., 2011a). These approaches are constrained by the number of genotypes that can be tested: therefore, as with LTT, we recommend that an informative subset of genotypes be used to empirically assess vernalization response within a framework of complete monitoring of environmental conditions.

### Insights Into LTT and VRN Sensitivity From GWAS

The large panel of accessions used in this study (Table 2 and Figure 5A) showed that the most significant determinants of WS were *FR-H1* and *FR-H2*, confirming previous work (reviewed by Cuesta-Marcos et al., 2015). Implications of this finding range from the trivial to the exciting. The large number of accessions with WS values within 20% of the spring check (Figure 1A) provides the trivial explanation: despite the stated criteria that all submissions to the panel have maximum LTT, additional phenotypic and genotypic pre-screening may have been advisable. For example, every accession in the top 5% for LTT has the complete intron in *VRN-H1* (Table 3), suggesting that this haplotype could have been a useful criterion for inclusion of accessions in the GWAS panel. The exciting implication of the finding that *FR-H1* and *FR-H2* are significant sources of variation is that there is much more to be learned about allelic variation at these loci, using locus in the broadest sense of the term. For example, *FR-H2* is a complex locus, where copy number variation (CNV) in CBF gene family members is a driver of differences in degree of LTT (Stockinger et al., 2007; Knox et al., 2010; Francia et al., 2016). The significant effect of this complex locus on LTT could, therefore, represent an example of the importance of CNV in barley, a topic addressed in general by Muñoz-Amatriaín et al. (2013), and with specific reference to *FR-H2* by Muñoz-Amatriaín and Mascher (2018). In the case of *FR-H1*, there are additional genes in physical proximity with *HvBM5A* whose annotation makes them intriguing possible determinants of LTT (Cuesta-Marcos et al., 2015). Allelic variation at this “super gene” complex could, therefore, account for the continued importance of this QTL.

The discovery of candidate genes for *FR-H3* in this GWAS panel is a key finding. While all three genes merit additional investigation, and two or more of them could be acting in concert as a complex locus, *HORVU1Hr1G012710* is the most obvious candidate, based on annotation. This gene encodes barley lipid transfer protein 101 (*blt101*), which is known to be induced by low temperatures (Brown et al., 2001) and may be involved in the differential WS of barley genotypes by slowing growth under low temperatures and increasing tolerance to water stress (Choi and Hwang, 2015). Choi and Hwang (2015) also showed that transgenic wheat overexpressing *blt101* had greater LTT than the wild type. *HORVU1Hr1G012680* encodes a protein in the UDP-glycosyltransferase superfamily. Members of this family are reported to be involved in LTT via flavonoid glycosylation (Schulz et al., 2016; Zhao et al., 2019). *HORVU1Hr1G012690*, which contains the most significantly associated SNP (SCRI_RS_114047), encodes a tetraspanin family protein. Tetraspanins are small transmembrane proteins with important roles in plant development and responses to biotic and abiotic stresses, including low temperatures (reviewed by Reimann et al., 2017).

The significant effect of *VRN-H2* on LTT in this study is likely due to the prevalence of winter types with *VRN-H2* and high LTT and accessions lacking *VRN-H2* with low LTT (Figure 2C). This argument for non-causative correlation of *VRN-H2* with LTT is supported by the equal WS values for Alba (winter check) and Maja (facultative check) (Figure 1A) and the presence of the facultative accessions in the top 5% with *VRN-H2* deletions (Table 3). The significant main effect of *PPD-H1* on LTT requires additional research. Vegetative stage low temperature injury is expected under field conditions to occur during winter, when there are short photoperiod conditions. However, there are reports involving differential LTT in controlled environment studies using long photoperiods (Stockinger et al., 2007; Dhillon et al., 2010).

In addition to identifying *FR-H1*, *FR-H2*, *FR-H3*, *VRN-H2*, and *PPD-H1*, the GWAS meta-analysis of WS revealed additional novel loci that merit further investigation (Table 2 and Supplementary Table S5). Most of these loci were not significant in any of the individual environments, supporting the power of the meta-analysis, which is used in human medicine for the identification of genetic risk factors for complex diseases (Evangelou and Ioannidis, 2013). By combining the results from different GWAS to increase sample size, this approach increases statistical power and reduces false positives (Wang and Xu, 2019). No WS QTLs were detected using the meta-analysis, or in the GWAS of individual environment data, in the vicinity of the *Vrs1* locus on chromosome 2H. This locus is the primary determinant of row type in barley (Komatsuda et al., 2007), and its lack of effect on LTT supports the hypothesis that the association of the 6-row phenotype and high LTT in this study (Figures 2D, 5A) is due to unintentional bias in choosing accessions for inclusion. At the time the study was designed, the largest contributors of germplasm were breeding programs focusing on 6-rows. Given the current predominance of 2-row barley for malting and brewing, future LTT research should focus on this row type. Furthermore, our data suggest that the WS of 6-row barley can be transferred to 2-row barley.

With a complex trait, such as LTT, interactions will likely be the rule rather than the exception. We found significant two-locus interactions for *VRN-H1*, *VRN-H2*, and *PPD-H1* with *FR-H2* and novel QTLs. The lack of significant interaction between *VRN-H1* and *VRN-H2* for WS at any test site is further evidence that specific combinations of alleles at these loci are not required for maximum LTT—an encouraging prospect for the potential of facultative growth habit. Understanding the interaction of *PPD-H1* with *FR-H2* will require further research. The results of these investigations could lead to the addition of *PPD-H1* as a criterion for facultative growth habit.

The GWAS of DTF under greenhouse conditions (Figure 5B) identified the principal determinants of vernalization response reported in the literature: *VRN-H1* and *VRN-H2* (reviewed by Cuesta-Marcos et al., 2015). Furthermore, these loci showed the largest two-locus interaction we detected in this study, as expected based on the model originally proposed by Takahashi and Yasuda (1971) and subsequently validated by Yan et al. (2003) and Szũcs et al. (2007). Interestingly, *VRN-H3* was not a determinant of DTF in this array: either because of monomorphism in the germplasm or the environmental conditions of the greenhouse assay. Loscos et al. (2014) characterized CNVs at this locus in barley germplasm and identified associations with DTF. Extending this research to the current germplasm, or subsets thereof, is warranted. The significance of *PPD-H1* in VRN sensitivity (Figure 5B) may be attributable to the long-day photoperiod (ld-PPD, 16 h light/8 h dark) used in the controlled environment assay. We also observed a significant interaction of *PPD-H1* and *VRN-H2*, as reported in other germplasm (Laurie et al., 1995; Karsai et al., 2005; Turner et al., 2013), providing additional evidence for the interconnectedness of the vernalization and photoperiod pathways under long-day conditions.

### Additional Insights From the Top 5% for LTT

The top 5% (Tables 1, 3 and Figure 3) will be a resource for introgressing alleles contributing to maximum LTT into the current barley germplasm and for deeper analysis of the genetics and physiology of LTT. Most of these accessions are 6-rows, and most are feed barley cultivars or land race accessions. Therefore, targeted allele introgression into elite 2-row malting germplasm will be required. The prevalence of the sd-PPD sensitivity allele at *PPD-H2* in the top 5% is notable and suggests a fitness advantage to this phenotype, in the absence of direct selection. We hypothesize that significant QTL effects for this locus on LTT were not detected in the current data set because mid-winter warm temperature events did not occur in the test environments. The sd-PPD sensitivity phenotype, therefore, can be viewed as an insurance mechanism. Validation of its value will require controlled environment experiments and “fortuitous” field trials when winter warm temperature events occur. The presence of the sd-PPD insensitive allele in accessions with winter growth habit may be attributable to the historical dependency on VRN sensitivity to delay the vegetative to reproductive transition and/or to its importance in winter barleys from regions with mild winters (Casao et al., 2011b)

The prevalence of the ld-PPD sensitivity allele at *PPD-H1* in all of the top 5% could be due to selection for early maturity in fall-planted barley and not to a direct effect on LTT. The widespread occurrence of the ld-PPD insensitive allele in spring habit barley is credited with enhancing yield by extending the grain-filling period (Turner et al., 2005). Introgressing the ld-PPD insensitive into fall-planted germplasm could, therefore, be one path to increasing grain yield. There is evidence of selection for this allele in germplasm from Southern Europe (Casao et al., 2011b).

### Prospects for Facultative Growth Habit

Climate change makes it imperative to ensure that crops are resilient in the face of volatility in temperature and precipitation. Facultative varieties offer one path forward for barley: the capacity to acclimate and achieve maximum LTT without vernalization sensitivity, coupled with the “insurance of sd-PPD sensitivity,” will allow for flexibility in planting date. There is precedent in the literature that facultative growth habit types are capable of achieving the same LTT as winter growth habit types (Fowler et al., 2001; von Zitzewitz et al., 2011; Fisk et al., 2013; Rizza et al., 2016). In the current research, the facultative check Maja had a WS value comparable to the winter check Alba. The current GWAS panel was assembled to study LTT irrespective of growth habit. Therefore, it is encouraging that facultative types were among the top 5% for LTT. Marker assisted selection for loss-of-function alleles at *VRN-H2* at *PPD-H2* now provides an efficient path to target facultative growth habit. Rigorous assessment of the prospects for this growth habit type will (1) require extensive assessment of larger numbers of diverse facultative accessions, compared with winter checks, in multiple environments and (2) be facilitated by the development and deep characterization of isogenic lines developed through traditional introgression or, potentially, through genome editing.

## Data Availability Statement

The datasets generated for this study can be found in the online repositories. The names of the repository/repositories and accession number(s) can be found in the article/ Supplementary Material.

## Author Contributions

AC-M, PH, DH, SF, and LH designed the experiment, provided seed to the cooperators, curated the data, and conducted the preliminary analyses. MM-A and JH had primary responsibility for data analysis. JH, MM-A, and PH prepared the drafts, edited the drafts, and ensured co-author access to the evolving manuscript. Co-authors PH, FC, CE, AG, MH, GH, EI, IK, TN, KeS, ES, and WT grew the experiment and generated the WS data, they and all the other co-authors participated in reviewing and editing. All authors read and approved the manuscript.

## Conflict of Interest

AB was employed by the company Limagrain Europe, AC-M was employed by Bayer Crop Science, CE was employed by Saatzucht Ackermann GmbH & Co. KG, and AG was employed by the company Secobra Recherches. The remaining authors declare that the research was conducted in the absence of any commercial or financial relationships that could be construed as a potential conflict of interest.

## Footnotes

1 https://www.ars-grin.gov/

2 http://barlex.barleysequence.org/

## References

[B1] Belcher A. R. Graebner R. C. Cuesta-Marcos A. Fisk S. Filichkin T. Smith K. P. (2015). Registration of the TCAP FAC-WIN6 barley panel for genomewide association studies. *J. Plant Regist.* 9 411–418. 10.3198/jpr2014.12.0083crmp

[B2] Benjamini Y. Hochberg Y. (1995). Controlling the false discovery rate: a practical and powerful approach to multiple testing. *J. R. Stat. Soc. Ser. B* 57 289–300. 10.1111/j.2517-6161.1995.tb02031.x

[B3] Bradbury P. J. Zhang Z. Kroon D. E. Casstevens T. M. Ramdoss Y. Buckler E. S. (2007). TASSEL: software for association mapping of complex traits in diverse samples. *Bioinformatics* 23 2633–2635. 10.1093/bioinformatics/btm308 17586829

[B4] Brown A. P. Dunn M. A. Goddard N. J. Hughes M. A. (2001). Identification of a novel low-temperature-response element in the promoter of the barley (*Hordeum vulgare* L) gene blt101.1. *Planta* 213 770–780. 10.1007/s004250100549 11678282

[B5] Byrns B. M. Greer K. J. Fowler D. B. (2020). Modelling winter survival in cereals: an interactive tool. *Crop. Sci.* 60 2408–2419. 10.1002/csc2.20246

[B6] Casao M. C. Igartua E. Karsai I. Lasa J. M. Gracia M. P. Casas A. M. (2011a). Expression analysis of vernalization and day-length response genes in barley (*Hordeum vulgare* L.) indicates that VRNH2 is a repressor of PPDH2 (HvFT3) under long days. *J. Exp. Bot.* 62 1939–1949. 10.1093/jxb/erq382 21131547 PMC3060678

[B7] Casao M. C. Karsai I. Igartua E. Gracia M. P. Veisz O. Casas A. M. (2011b). Adaptation of barley to mild winters: a role for PPDH2. *BMC Plant Biol.* 11:164 10.1186/1471-2229-11-164 PMC3226555 22098798

[B8] Chen A. Gusta L. V. Brûlé-Babel A. Leach R. Baumann U. Fincher G. B. (2009). Varietal and chromosome 2H locus-specific frost tolerance in reproductive tissues of barley (*Hordeum vulgare* L.) detected using a frost simulation chamber. *Theor. Appl. Genet.* 119 685–694. 10.1007/s00122-009-1079-1 19484216

[B9] Choi C. Hwang C. H. (2015). The barley lipid transfer protein, BLT101, enhances cold tolerance in wheat under cold stress. *Plant Biotechnol. Rep.* 9 197–207. 10.1007/s11816-015-0357-4

[B10] Cockram J. Jones H. Leigh F. J. O’Sullivan D. Powell W. Laurie D. A. (2007). Control of flowering time in temperate cereals: genes, domestication, and sustainable productivity. *J. Exp. Bot.* 58 1231–1244. 10.1093/jxb/erm042 17420173

[B11] Cockram J. White J. Zuluaga D. L. Smith D. Comadran J. Macaulay M. (2010). Genome-wide association mapping to candidate polymorphism resolution in the unsequenced barley genome. *Proc. Natl. Acad. Sci. U.S.A.* 107:21611 10.1073/pnas.1010179107 PMC3003063 21115826

[B12] Colmsee C. Beier S. Himmelbach A. Schmutzer T. Stein N. Scholz U. (2015). BARLEX - the barley draft genome explorer. *Mol. Plant* 8 964–966. 10.1016/j.molp.2015.03.009 25804976

[B13] Comadran J. Kilian B. Russell J. Ramsay L. Stein N. Ganal M. (2012). Natural variation in a homolog of Antirrhinum centroradialis contributed to spring growth habit and environmental adaptation in cultivated barley. *Nat. Genet.* 44:1388 10.1038/ng.2447 23160098

[B14] Cuesta-Marcos A. Casas A. M. Yahiaoui S. Gracia M. P. Lasa J. M. Igartua E. (2008). Joint analysis for heading date QTL in small interconnected barley populations. *Mol. Breed.* 21 383–399. 10.1007/s11032-007-9139-1

[B15] Cuesta-Marcos A. Muñoz-Amatriaín M. Filichkin T. Karsai I. Trevaskis B. Yasuda S. (2015). The relationships between development and low temperature tolerance in barley near isogenic lines differing for flowering behavior. *Plant Cell Physiol.* 56 2312–2324. 10.1093/pcp/pcv147 26443377

[B16] Cuesta-Marcos A. Szûcs P. Close T. J. Filichkin T. Muehlbauer G. J. Smith K. P. (2010). Genome-wide SNPs and re-sequencing of growth habit and inflorescence genes in barley: implications for association mapping in germplasm arrays varying in size and structure. *BMC Genomics* 11:707 10.1186/1471-2164-11-707 PMC3018479 21159198

[B17] Danyluk J. Kane N. A. Breton G. Limin A. E. Fowler D. B. Sarhan F. (2003). TaVRT-1, a putative transcription factor associated with vegetative to reproductive transition in cereals. *Plant Physiol.* 132 1849–1860. 10.1104/pp.103.023523 12913142 PMC181271

[B18] Dhillon T. Pearce S. P. Stockinger E. J. Distelfeld A. Li C. Knox A. K. (2010). Regulation of freezing tolerance and flowering in temperate cereals: the VRN-1 connection. *Plant Physiol.* 153 1846–1858. 10.1104/pp.110.159079 20571115 PMC2923912

[B19] Evangelou E. Ioannidis J. P. A. (2013). Meta-analysis methods for genome-wide association studies and beyond. *Nat. Rev. Genet.* 14 379–389. 10.1038/nrg3472 23657481

[B20] Faure S. Higgins J. Turner A. Laurie D. A. (2007). The flowering locus T-like gene family in barley (*Hordeum vulgare*). *Genetics* 176 599–609. 10.1534/genetics.106.069500 17339225 PMC1893030

[B21] Fisher R. (1925). *Statistical Methods for Research Workers*, 1st Edn Edinburgh: Oliver & Boyd.

[B22] Fisk S. P. Cuesta-Marcos A. Cistué L. Russell J. Smith K. P. Baenziger S. (2013). FR-H3: a new QTL to assist in the development of fall-sown barley with superior low temperature tolerance. *Theor. Appl. Genet.* 126 335–347. 10.1007/s00122-012-1982-8 23052020

[B23] Fowler D. B. Breton G. Limin A. E. Mahfoozi S. Sarhan F. (2001). Photoperiod and temperature interactions regulate low-temperature-induced gene expression in barley. *Plant Physiol.* 127 1676–1681. 10.1104/pp.010483 11743112 PMC133572

[B24] Francia E. Morcia C. Pasquariello M. Mazzamurro V. Milc J. A. Rizza F. (2016). Copy number variation at the HvCBF4-HvCBF2 genomic segment is a major component of frost resistance in barley. *Plant Mol. Biol.* 92 161–175. 10.1007/s11103-016-0505-4 27338258

[B25] Fu D. Szucs P. Yan L. Helguera M. Skinner J. S. von Zitzewitz J. (2005). Large deletions within the first intron in VRN-1 are associated with spring growth habit in barley and wheat. *Mol. Genet. Genomics* 273 54–65. 10.1007/s00438-004-1095-4 15690172

[B26] Hayes P. M. Blake T. Chen T. H. Tragoonrung S. Chen F. Pan A. (1993). Quantitative trait loci on barley (*Hordeum vulgare* L.) chromosome 7 associated with components of winterhardiness. *Genome* 36 66–71. 10.1139/g93-009 18469970

[B27] Igartua E. Casas A. M. Ciudad F. Montoya J. L. Romagosa I. (1999). RFLP markers associated with major genes controlling heading date evaluated in a barley germ plasm pool. *Heredity* 83 551–559. 10.1046/j.1365-2540.1999.00589.x 10620027

[B28] Kang H. (2015). Statistical considerations in meta-analysis. *Hanyang. Med. Rev.* 35 23–32. 10.7599/hmr.2015.35.1.23

[B29] Karsai I. Mészáros K. Szücs P. Hayes P. M. Láng L. Bedö Z. (1999). Effects of loci determining photoperiod sensitivity (Ppd-H1) and vernalization response (Sh2) on agronomic traits in the ‘Dicktoo’×‘Morex’ barley mapping population. *Plant Breed.* 118 399–403. 10.1046/j.1439-0523.1999.00408.x

[B30] Karsai I. Szũcs P. Köszegi B. Hayes P. M. Casas A. Bedõ Z. (2008). Effects of photo and thermo cycles on flowering time in barley: a genetical phenomics approach. *J. Exp. Bot.* 59 2707–2715. 10.1093/jxb/ern131 18550600 PMC2486468

[B31] Karsai I. Szũcs P. Mészáros K. Filichkina T. Hayes P. Skinner J. (2005). The Vrn-H2 locus is a major determinant of flowering time in a facultative× winter growth habit barley (*Hordeum vulgare* L.) mapping population. *Theor. Appl. Genet.* 110 1458–1466. 10.1007/s00122-005-1979-7 15834697

[B32] Kikuchi R. Kawahigashi H. Ando T. Tonooka T. Handa H. (2009). Molecular and functional characterization of PEBP genes in barley reveal the diversification of their roles in flowering. *Plant Physiol.* 149:1341 10.1104/pp.108.132134 PMC2649388 19168644

[B33] Kikuchi R. Kawahigashi H. Oshima M. Ando T. Handa H. (2012). The differential expression of HvCO9, a member of the CONSTANS-like gene family, contributes to the control of flowering under short-day conditions in barley. *J. Exp. Bot.* 63 773–784. 10.1093/jxb/err299 22016423 PMC3254679

[B34] Knox A. K. Dhillon T. Cheng H. Tondelli A. Pecchioni N. Stockinger E. J. (2010). CBF gene copy number variation at frost resistance-2 is associated with levels of freezing tolerance in temperate-climate cereals. *Theor. Appl. Genet.* 121 21–35. 10.1007/s00122-010-1288-7 20213518

[B35] Komatsuda T. Pourkheirandish M. He C. Azhaguvel P. Kanamori H. Perovic D. (2007). Six-rowed barley originated from a mutation in a homeodomain-leucine zipper I-class homeobox gene. *Proc. Natl. Acad. Sci. U.S.A.* 104 1424–1429. 10.1073/pnas.0608580104 17220272 PMC1783110

[B36] Laurie D. Pratchett N. Bezant J. Snape J. (1995). RFLP mapping of five major genes and eight quantitative trait loci controlling flowering time in a winter x spring barley (*Hordeum vulgare* L.) cross. *Genome* 38 575–585. 10.1139/g95-074 18470191

[B37] Lin C.-S. Poushinsky G. (1983). A modified augmented design for an early stage of plant selection involving a large number of test lines without replication. *Biometrics* 39 553–561. 10.2307/2531083

[B38] Lin C.-S. Poushinsky G. (1985). A modified augmented design (type 2) for rectangular plots. *Can. J. Plant Sci.* 65 743–749. 10.4141/cjps85-094

[B39] Loscos J. Igartua E. Contreras-Moreira B. Gracia M. P. Casas A. M. (2014). HvFT1 polymorphism and effect-survey of barley germplasm and expression analysis. *Front. Plant Sci.* 5:251 10.3389/fpls.2014.00251 PMC4047512 24936204

[B40] Manning A. K. LaValley M. Liu C.-T. Rice K. An P. Liu Y. (2011). Meta-analysis of gene-environment interaction: joint estimation of SNP and SNP × environment regression coefficients. *Genet. Epidemiol.* 35 11–18. 10.1002/gepi.20546 21181894 PMC3312394

[B41] Mascher M. Gundlach H. Himmelbach A. Beier S. Twardziok S. O. Wicker T. (2017). A chromosome conformation capture ordered sequence of the barley genome. *Nature* 544 427–433. 10.1038/nature22043 28447635

[B42] Muñoz-Amatriaín M. Eichten S. R. Wicker T. Richmond T. A. Mascher M. Steuernagel B. (2013). Distribution, functional impact, and origin mechanisms of copy number variation in the barley genome. *Genome Biol.* 14 R58 10.1186/gb-2013-14-6-r58 PMC3706897 23758725

[B43] Muñoz-Amatriaín M. Mascher M. (2018). “Sequence diversity and structural variation,” in *The Barley Genome. Compendium of Plant Genomes*, eds Stein N. Muehlbauer G. (Cham: Springer), 109–122. 10.1007/978-3-319-92528-8_8

[B44] Pan A. Hayes P. M. Chen F. Chen T. H. H. Blake T. Wright S. (1994). Genetic analysis of the components of winterhardiness in barley (s L.). *Theor. Appl. Genet.* 89 900–910. 10.1007/BF00224516 24178102

[B45] Powell N. Ji X. Ravash R. Edlington J. Dolferus R. (2012). Yield stability for cereals in a changing climate. *Funct. Plant Biol.* 39 539–552. 10.1071/fp12078 32480806

[B46] Reimann R. Kost B. Dettmer J. (2017). TETRASPANINs in plants. *Front. Plant Sci.* 8:545 10.3389/fpls.2017.00545 PMC5394113 28458676

[B47] Rizza F. Karsai I. Morcia C. Badeck F.-W. Terzi V. Pagani D. (2016). Association between the allele compositions of major plant developmental genes and frost tolerance in barley (*Hordeum vulgare* L.) germplasm of different origin. *Mol. Breed.* 36:156 10.1007/s11032-016-0571-y

[B48] Schulz E. Tohge T. Zuther E. Fernie A. R. Hincha D. K. (2016). Flavonoids are determinants of freezing tolerance and cold acclimation in *Arabidopsis thaliana*. *Sci. Rep.* 6:34027 10.1038/srep34027 PMC5034326 27658445

[B49] Skinner J. S. Szucs P. von Zitzewitz J. Marquez-Cedillo L. Filichkin T. Stockinger E. J. (2006). Mapping of barley homologs to genes that regulate low temperature tolerance in Arabidopsis. *Theor. Appl. Genet.* 112 832–842. 10.1007/s00122-005-0185-y 16365758

[B50] Skinner J. S. von Zitzewitz J. Szucs P. Marquez-Cedillo L. Filichkin T. Amundsen K. (2005). Structural, functional, and phylogenetic characterization of a large CBF gene family in barley. *Plant Mol. Biol.* 59 533–551. 10.1007/s11103-005-2498-2 16244905

[B51] Stockinger E. J. Skinner J. S. Gardner K. G. Francia E. Pecchioni N. (2007). Expression levels of barley Cbf genes at the Frost resistance-H2 locus are dependent upon alleles at Fr-H1 and Fr-H2. *Plant J.* 51 308–321. 10.1111/j.1365-313X.2007.0141.x 17559507

[B52] Szũcs P. Skinner J. S. Karsai I. Cuesta-Marcos A. Haggard K. G. Corey A. E. (2007). Validation of the VRN-H2/VRN-H1 epistatic model in barley reveals that intron length variation in VRN-H1 may account for a continuum of vernalization sensitivity. *Mol. Genet. Genomics* 277 249–261. 10.1007/s00438-006-0195-8 17151889

[B53] Takahashi R. Yasuda S. (1971). “Genetics of earliness and growth habit in barley,” in *Proceedings of the 2nd International Barley Genetics Symposium*, ed. Nilan R. (Washington, DC: Washington State University Press), 388–408.

[B54] Tondelli A. Xu X. Moragues M. Sharma R. Schnaithmann F. Ingvardsen C. (2013). Structural and temporal variation in genetic diversity of european spring two-row barley cultivars and association mapping of quantitative traits. *Plant Genome* 6:Plantgenome2013.2003.0007 10.3835/plantgenome2013.03.0007

[B55] Trevaskis B. Bagnall D. J. Ellis M. H. Peacock W. J. Dennis E. S. (2003). MADS box genes control vernalization-induced flowering in cereals. *Proc. Natl. Acad. Sci. U.S.A.* 100:13099 10.1073/pnas.1635053100 PMC240751 14557548

[B56] Trevaskis B. Tadege M. Hemming M. N. Peacock W. J. Dennis E. S. Sheldon C. (2007). Short vegetative phase-like MADS-box genes inhibit floral meristem identity in barley. *Plant Physiol.* 143 225–235. 10.1104/pp.106.090860 17114273 PMC1761976

[B57] Turner A. Beales J. Faure S. Dunford R. P. Laurie D. A. (2005). The pseudo-response regulator Ppd-H1 provides adaptation to photoperiod in barley. *Science* 310 1031–1034. 10.1126/science.1117619 16284181

[B58] Turner A. S. Faure S. Zhang Y. Laurie D. A. (2013). The effect of day-neutral mutations in barley and wheat on the interaction between photoperiod and vernalization. *Theor. Appl. Genet.* 126 2267–2277. 10.1007/s00122-013-2133-6 23737074 PMC3755224

[B59] von Zitzewitz J. Cuesta-Marcos A. Condon F. Castro A. J. Chao S. Corey A. (2011). The genetics of winterhardiness in barley: perspectives from genome-wide association mapping. *Plant Genome* 4 76–91. 10.3835/plantgenome2010.12.0030

[B60] von Zitzewitz J. Szûcs P. Dubcovsky J. Yan L. Francia E. Pecchioni N. (2005). Molecular and structural characterization of barley vernalization genes. *Plant Mol. Biol.* 59 449–467. 10.1007/s11103-005-0351-2 16235110

[B61] Wang M. Xu S. (2019). Statistical power in genome-wide association studies and quantitative trait locus mapping. *Heredity* 123 287–306. 10.1038/s41437-019-0205-3 30858595 PMC6781134

[B62] Yan L. Fu D. Li C. Blechl A. Tranquilli G. Bonafede M. (2006). The wheat and barley vernalization gene VRN3 is an orthologue of FT. *Proc. Natl. Acad. Sci. U.S.A.* 103:19581 10.1073/pnas.0607142103 PMC1748268 17158798

[B63] Yan L. Loukoianov A. Blechl A. Tranquilli G. Ramakrishna W. SanMiguel P. (2004). The wheat VRN2 gene is a flowering repressor down-regulated by vernalization. *Science* 303 1640–1644. 10.1126/science.1094305 15016992 PMC4737501

[B64] Yan L. Loukoianov A. Tranquilli G. Helguera M. Fahima T. Dubcovsky J. (2003). Positional cloning of the wheat vernalization gene VRN1. *Proc. Natl. Acad. Sci. U.S.A.* 100:6263 10.1073/pnas.0937399100 PMC156360 12730378

[B65] Zhang Z. Ersoz E. Lai C.-Q. Todhunter R. J. Tiwari H. K. Gore M. A. (2010). Mixed linear model approach adapted for genome-wide association studies. *Nat. Genet.* 42 355–360. 10.1038/ng.546 20208535 PMC2931336

[B66] Zhao M. Jin J. Gao T. Zhang N. Jing T. Wang J. (2019). Glucosyltransferase CsUGT78A14 regulates flavonols accumulation and reactive oxygen species scavenging in response to cold stress in *Camellia sinensis*. *Front. Plant Sci.* 10:1675 10.3389/fpls.2019.01675 PMC6941654 31929783

