# Supplementary material for: Perspectives on Low Temperature Tolerance and Vernalization Sensitivity in Barley: Prospects for Facultative Growth Habit
Source: Front Plant Sci. 2020 Nov 9;11:585927. doi: 10.3389/fpls.2020.585927 (PMC7814503; doi:10.3389/fpls.2020.585927)
Supplement: Supplementary file 7 [file Presentation_1.PPTX]

## Slide 1
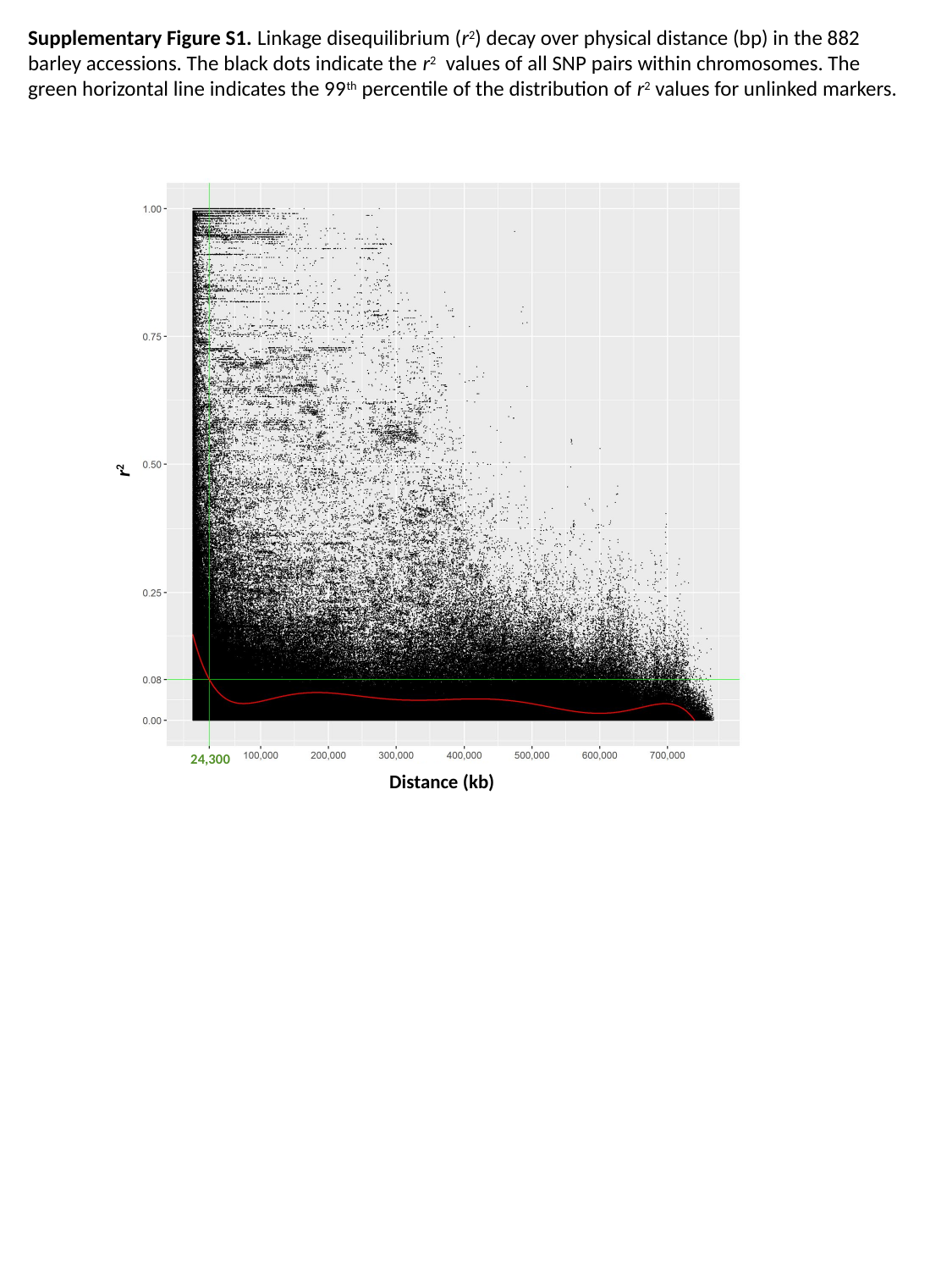

Supplementary Figure S1. Linkage disequilibrium (r2) decay over physical distance (bp) in the 882 barley accessions. The black dots indicate the r2 values of all SNP pairs within chromosomes. The green horizontal line indicates the 99th percentile of the distribution of r2 values for unlinked markers.
r2
24,300
Distance (kb)
