# Supplementary material for: Perspectives on Low Temperature Tolerance and Vernalization Sensitivity in Barley: Prospects for Facultative Growth Habit
Source: Front Plant Sci. 2020 Nov 9;11:585927. doi: 10.3389/fpls.2020.585927 (PMC7814503; doi:10.3389/fpls.2020.585927)
Supplement: Supplementary file 8 [file Presentation_2.PPTX]

## Slide 1
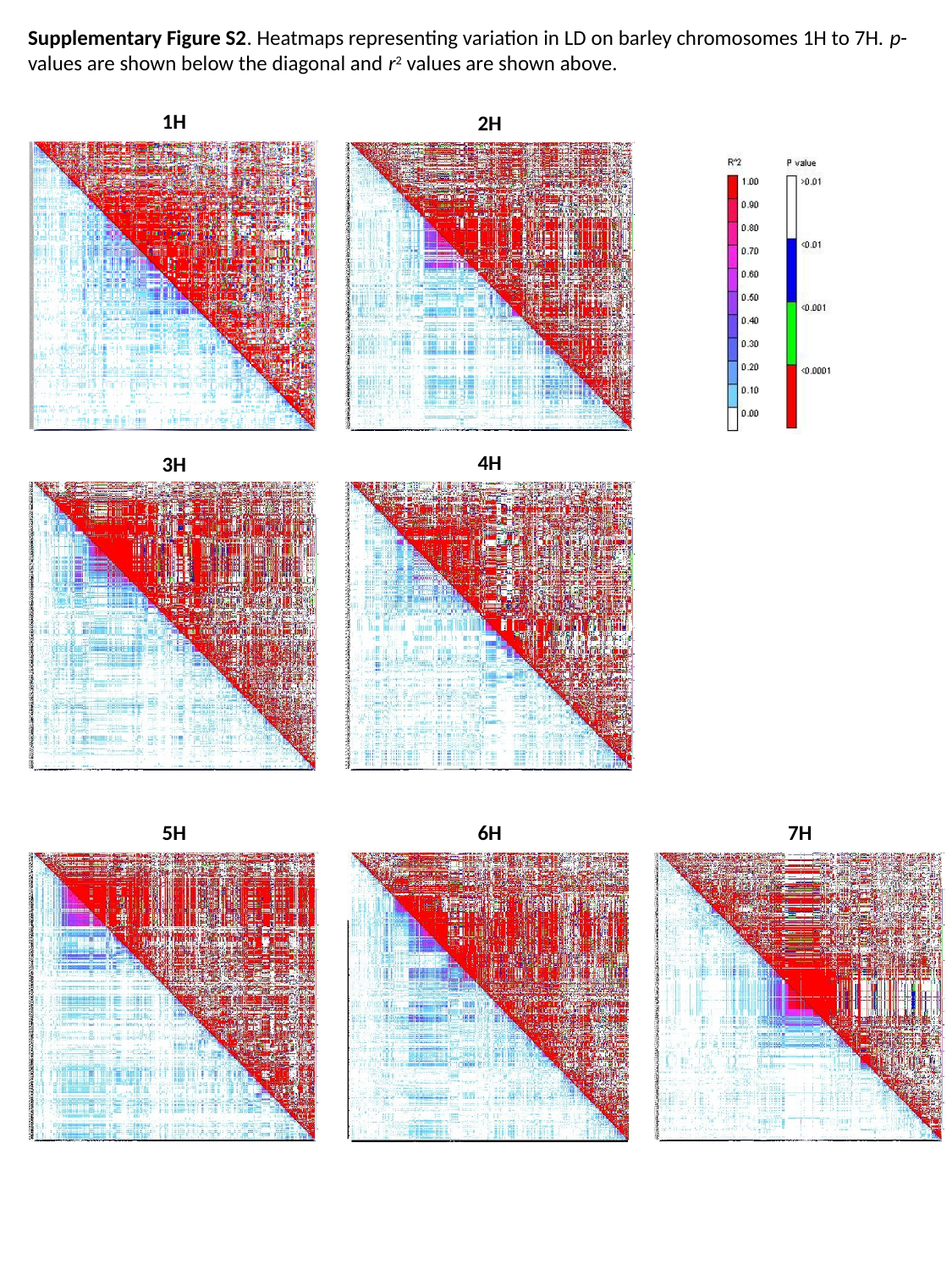

Supplementary Figure S2. Heatmaps representing variation in LD on barley chromosomes 1H to 7H. p-values are shown below the diagonal and r2 values are shown above.
1H
2H
4H
3H
6H
7H
5H
